# Comparative Transcriptomic Analysis of Largemouth Bass (*Micropterus salmoides*) Livers Reveals Response Mechanisms to High Temperatures

**DOI:** 10.3390/genes14112096

**Published:** 2023-11-17

**Authors:** Fan Zhou, Ming Qi, Jiapeng Li, Yuanfei Huang, Xiaoming Chen, Wei Liu, Gaohua Yao, Qinghui Meng, Tianlun Zheng, Zhanqi Wang, Xueyan Ding

**Affiliations:** 1 Zhejiang Fisheries Technical Extension Center, Hangzhou 310023, China; zhoufan0302@126.com (F.Z.); qiming_1120@163.com (M.Q.); 13567189845@163.com (Y.H.); 13738033504@163.com (X.C.); zjscliuwei@163.com (W.L.); 15988855806@163.com (G.Y.); qinghui1234@126.com (Q.M.); tlzheng@sohu.com (T.Z.); 2 Key Laboratory of Vector Biology and Pathogen Control of Zhejiang Province, College of Life Sciences, Huzhou University, Huzhou 313000, China; ljpbangbangde@163.com

**Keywords:** *M. salmoides*, liver, heat stress, transcriptome, response mechanism

## Abstract

High temperatures are considered one of the most significant limitations to subtropical fishery production. Largemouth bass (*Micropterus salmoides*) is an economically important freshwater species grown in subtropical areas, which are extremely sensitive to heat stress (HS). However, comprehensive transcriptomic data for the livers of largemouth bass in response to HS are still lacking. In this study, a comparative transcriptomic analysis was performed to investigate the gene expression profiles of the livers of largemouth bass under HS treatment. As a result, 6114 significantly differentially expressed genes (DEGs), which included 2645 up-regulated and 3469 down-regulated genes, were identified in response to HS. Bioinformatics analyses demonstrated that the ‘ECM-receptor interaction’ pathway was one of the most dramatically changed pathways in response to HS, and eight DEGs assigned to this pathway were taken as hub genes. Furthermore, the expression of these eight hub genes was determined by quantitative reverse transcription PCR, and all of them showed a significant change at the transcriptional level, suggesting a crucial role of the ‘ECM-receptor interaction’ pathway in the response of largemouth bass to HS. These findings may improve our understanding of the molecular mechanisms underlying the response of largemouth bass to HS.

## 1. Introduction

Climate change is one of the main environmental factors affecting the survival of plants, animals, humans, and other living organisms [1]. Owing to the ongoing intensification of global warming, extreme temperature events have become more frequent and severe in recent years, posing a serious threat to the aquaculture industry [2,3]. Most fish are ectotherms and must adapt to temperature changes through biochemical, physiological, and morphological plasticities and/or microevolution [4,5]. When fish are subjected to rapid temperature alterations, the expression of an array of genes with vital roles in stress response, cell survival, and maintenance is induced [6,7,8]. However, when water temperatures exceed the thermal tolerance limits of fish, normal growth, development, survival, and reproductive performance are significantly affected [9,10]. In addition, elevated temperatures facilitate the movement of pathogens, leading to a significant increase in disease outbreaks and economic losses in aquaculture systems [11,12].

Previous studies have indicated that high-temperature stress, also known as heat stress (HS), can cause many adverse effects on fish, such as reduced antioxidase activity, decreased metabolic activity, and the activation of cell apoptosis [6,13,14]. HS also increases the production of reactive oxygen species (ROS), induces oxidative stress, and activates signal transduction [13,15,16]. As a result of the long-term coevolution, fish have evolved several conserved strategies to cope with HS [17,18]. Fish can initiate a variety of antioxidant enzymes to eliminate excess ROS [6,19]. In addition to the antioxidant defense system, heat shock protein (HSP)-mediated stress response (HSR) is another crucial strategy for managing HS [16,20]. HSR is conserved across kingdoms and can minimize adverse effects and restore cellular homeostasis [16,19,21]. Additionally, mitogen-activated protein kinase (MAPK)-mediated signal transduction pathways have also been reported to positively respond to HS [16,22]. Although these findings are encouraging, further research is required to elucidate the molecular mechanisms underlying the regulation of fish responses to HS.

Largemouth bass (*M. salmoides*), which is naturally distributed in North America, has spread worldwide as a commercial aquaculture species [5,23,24,25]. In the early 1980s, largemouth bass was introduced to Guangdong Province in Southern China, and it is now a very important and economically valuable freshwater species, with an annual production of more than 0.7 million tons in 2021 [24]. Largemouth bass is a subtropical fish species that grows at optimum temperatures between 25 and 30 °C, whereas temperatures exceeding 30 °C affect the normal growth of these fish [5,12,26]. Furthermore, when temperatures exceed 30 °C, largemouth bass are highly susceptible to pathogens [12,27]. Over the past two decades, with the intensification of global warming, largemouth bass have suffered different degrees of HS and yield losses [5,12,26,28]. Thus, it is essential to explore the molecular mechanisms underlying the regulation of the largemouth bass response to HS.

RNA-sequencing (RNA-Seq)-based transcriptomic analysis is an indispensable method for extensively exploring gene expression profiles, identifying differentially expressed genes (DEGs), and providing integrated information regarding individual DEGs in growth, development, and stress responses [29]. In this study, a comparative transcriptomic analysis of the livers of largemouth bass was performed to explore the mechanisms underlying the response of largemouth bass to HS at the molecular level. Based on these measurements, 6114 DEGs were identified. Further bioinformatics analyses showed that ‘ECM-receptor interaction’ pathway-related genes are significantly changed following HS. In addition, the mRNA levels of the eight hub genes mapped to this pathway were further determined by quantitative reverse transcription PCR (qRT-PCR). All the hub genes were significantly altered at the transcriptional level in the livers of largemouth bass following HS. These findings provide new insights into the molecular mechanisms underlying the largemouth bass response to HS.

## 2. Materials and Methods

### 2.1. Animal Materials and Rearing Conditions

Largemouth bass (*M. salmoides*) cultivar ‘Zhelu No.1′, bred and preserved at the Zhejiang Fisheries Technical Extension Center (Hangzhou, China), was used. All fish obtained from this breeding farm were juveniles with a body length of 129.30 ± 7.66 mm and a body weight of 28.07 ± 5.00 g. Before the formal experiments, the fish were reared in 150 L tanks at a water temperature of 28 °C in the laboratory and fed a commercial bass diet (Hubei Haid Feeds Co., Ltd., Wuhan, China) as described previously [25,30]. After two weeks of acclimation, healthy largemouth bass were subjected to HS treatment.

### 2.2. HS Treatment and Sample Collection

The liver of fish is one of the most important organs and plays crucial roles in nutrient absorption, metabolism, and immune responses, as it is in charge of transforming toxic substances in the body. Furthermore, due to its susceptible nature, the liver is considered a critical target organ for environmental stimuli [31,32]. Thus, the liver of largemouth bass was used in this study. A total of 120 acclimatized largemouth bass were randomly divided into two groups of 60 fish each. The control group (Con) was reared at 28 °C. The HS group was reared at an initial water temperature of 28 °C, which was then increased to 38 °C at a rate of 1 °C/h for 8 h, 0.4 °C/h for 1 h, 0.2 °C/h for 8 h, and finally maintained at 38 °C for 7 h. During HS treatment, the first 18 fish that turned belly-up were defined as the heat-sensitive (HES) subgroup, and the last 18 fish that turned belly-up or survived were defined as the heat-tolerant (HET) subgroup. The experiments were repeated three times for each treatment under laboratory conditions, with six fish in each subset. After HS treatment, the largemouth bass was anesthetized with eugenol (Sigma-Aldrich, Munich, Germany), and the liver tissues were collected from the Con and HS-treated fish and immediately frozen and stored at −70 °C.

### 2.3. RNA Isolation, Library Construction and Illumina Sequencing

Total RNA was isolated from the livers of largemouth bass using TRIzol™ reagent (Invitrogen, Carlsbad, CA, USA). RNA quality was measured using a 5300 Bioanalyzer (Agilent Technologies, Santa Clara, CA, USA) and quantified using an ND-2000 spectrophotometer (NanoDrop Technologies, Wilmington, DE, USA). High-quality RNA samples were used for subsequent library construction. Liver sequencing libraries were constructed using a SuperScript double-stranded cDNA synthesis kit (Invitrogen) following the manufacturer’s instructions. After end repair, phosphorylation, and ‘A’ base addition, the cDNA libraries were analyzed on the Illumina NovaSeq 6000 sequencing system (Illumina, San Diego, CA, USA).

### 2.4. Transcriptome Assembly and DEG Screening

Raw sequencing reads were quality-trimmed using Fastp (v.0.19.5) [33] to get rid of low-quality reads, as described by Zhang et al. [32]. The clean reads were aligned to the reference genome of largemouth bass (NCBI accession number: GCF_014851395.1) [34] using HISAT2 (v.2.1.0) [35] in the orientation mode. Then, the mapped reads were assembled using StringTie [36] with the largemouth bass genome. For differential expression analysis, the fragments per kilobase of transcript per million fragments mapped (FPKM) at the gene level were calculated using RSEM (v.1.3.3) [37], and genes with a threshold of FPKM > 0.1 were taken as expressed [38,39]. For significant difference analysis, strict statistical thresholds with an absolute value of Log_2_FC (fold change) ≥ 1.0 and a Benjamini–Hochberg (BH)-adjusted *p*-value (Padj) < 0.05 were used as described previously [32,40].

### 2.5. Functional Annotation and Enrichment Analyses

Functional annotation analysis of the assembled genes was performed using Diamond (v.0.8.22), as described previously [40]. Gene Ontology (GO) and Kyoto Encyclopedia of Genes and Genomes (KEGG) enrichment analyses of DEGs between different pairwise comparisons were carried out using GOATOOLS [41] and KOBAS [42], respectively. The results of the enrichment analyses were visualized using the Majorbio Cloud platform (https://cloud.majorbio.com/, accessed on 10 April 2023) [43].

### 2.6. Short Time-Series Expression Miner (STEM) and Gene–Gene Interaction (GGI) Analyses

For STEM analysis, the HS treatment periods and the calculated Log_2_ values of the expression levels (in FPKM) of HES or HET to the Con were utilized as horizontal and vertical coordinates, as described previously [40,44]. Based on the expression patterns of the DEGs, distinct and significant clusters were constructed, as described by Maire et al. [45]. GGI prediction of hub DEGs was carried out using the STRING database (v.12.0, https://cn.string-db.org/, accessed on 16 May 2023) [46], and the interaction network was visualized using Cytoscape software (v.3.10.0) [47].

### 2.7. RNA Isolation and qRT-PCR Analysis

Total RNA was isolated from the livers of largemouth bass as described above. The obtained RNA was digested with DNase I and synthesized into cDNA using a PrimeScript™ RT Reagent Kit (TaKaRa, Dalian, China) following the manufacturer’s instructions. Quantitative PCR was carried out on a LightCycler^®^ 480 II instrument (Roche, Basel, Switzerland) using a TB Green^®^ Premix Ex Taq™ II Kit [48]. The relative mRNA expression levels were calculated using the 2^−ΔΔCT^ method [49], and the *β-actin* gene of largemouth bass was used as an internal control [50]. The primers used for qRT-PCR are listed in Table S1.

### 2.8. Statistical Analysis

Data were analyzed by one-way analysis of variance with Tukey’s test using SPSS software (version 27.0; SPSS Inc., Chicago, IL, USA). The results are given as the mean ± standard deviation (SD) of three biological replicates. Differences were considered statistically significant at a *p*-value of <0.05.

## 3. Results

### 3.1. Overview of Largemouth Bass Transcriptomes

**Figure 1:**
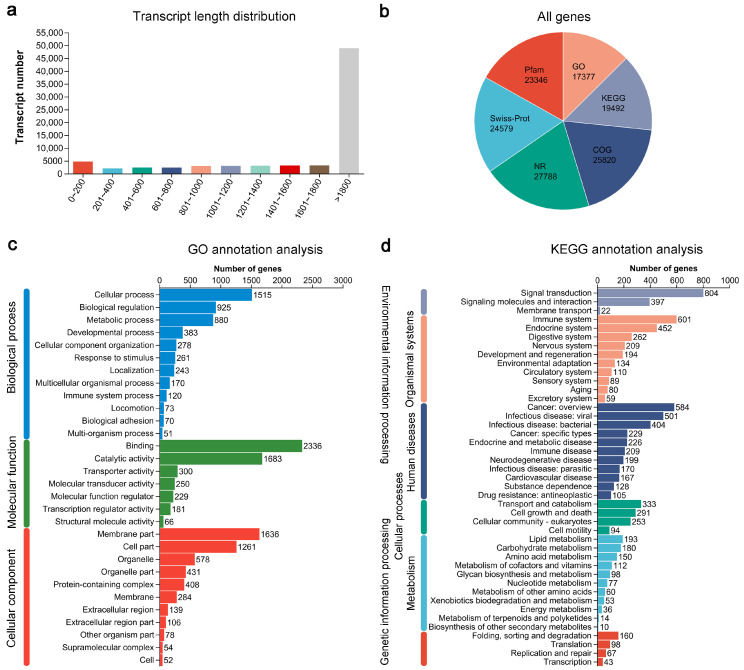
RNA-Seq of livers of largemouth bass (*M. salmoides*) following heat stress. (**a**) Length distribution of assembled transcripts in largemouth bass. (**b**) The number of genes annotated by different databases, including NCBI non-redundant protein (NR), Swiss-Prot, Gene Ontology (GO), Kyoto Encyclopedia of Genes and Genomes (KEGG), Protein Family (Pfam), and Clusters of Orthologous Group (COG) databases. (**c**) Classification of annotated GO terms. A total of 17,377 genes were annotated into 3 main classes: biological process (12 subclasses), molecular function (7 subclasses), and cellular component (11 subclasses). (**d**) Classification of annotated KEGG terms. A total of 19,492 genes were annotated into 6 main classes, including environmental information process (3 subclasses), organismal systems (10 subclasses), human diseases (11 subclasses), cellular processes (4 subclasses), metabolism (11 subclasses), and genetic information processing (4 subclasses).

To identify the responsive genes in largemouth bass following HS, the expression profiles of livers were analyzed. A total of 104.29 Gb of raw data was generated from 18 samples, of which 103.76 Gb was valid. After filtering, approximately 97.83% of the clean reads reached the threshold score of Q20, and approximately 93.30% reached Q30 (Table S2). The total and unique mapping rates were 96.89 and 82.92%, respectively (Table S2). Then, clean reads from the largemouth bass livers were aligned and assembled using HISAT2 (v.2.1.0) [35] and StringTie [36]. As shown in Figure 1a, the median and mean lengths of the transcripts were 2506 and 3154 bp, respectively. Many genes were annotated using several protein databases (Figure 1b). The most significant number of largemouth bass genes were annotated in the NCBI non-redundant protein (NR) database (27,788 genes), Clusters of Orthologous Genes (COG) database (25,820 genes), and Swiss-Prot database (24,579 genes).

To obtain more information about these largemouth bass genes, we conducted a functional classification using the terms annotated in the GO and KEGG databases. Consequently, 17,377 genes were assigned to at least one GO term. Regarding the biological process, ‘cellular process’, ‘biological regulation’, and ‘metabolic process’ were the three most abundant terms (Figure 1c). Regarding the molecular function, ‘binding’, ‘catalytic activity’, and ‘transporter activity’ were the three most represented terms (Figure 1c). Regarding the cellular component, ‘membrane part’, ‘cell part’, and ‘organelle’ were the three most enriched terms (Figure 1c). Additionally, 19,492 genes were assigned to various KEGG pathways, including those related to environmental information processing, organismal systems, human diseases, cellular processes, metabolism, and genetic information processing (Figure 1d). Collectively, these results suggest that HS treatment can lead to extensive gene rearrangements in the liver of largemouth bass.

### 3.2. Scanning of DEGs in Livers of Largemouth Bass in Response to HS

**Figure 2:**
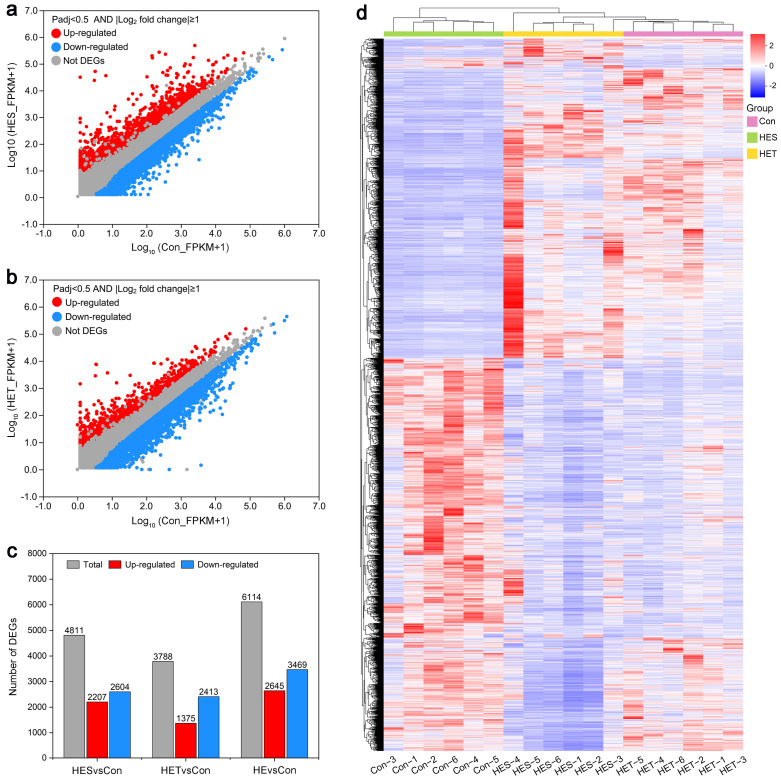
Transcriptional variation of largemouth bass (*M. salmoides*) exposed to high temperatures at different treatment points. (**a**,**b**) Differential expression analysis of differentially expressed genes (DEGs) in livers of largemouth bass of heat-sensitive (HES) subgroup (**a**) or heat-tolerant (HET) subgroup (**b**) by volcano plots. (**c**) The number of total, up-regulated, and down-regulated DEGs in the livers of largemouth bass following HES or HET treatment. (**d**) Expression analysis of the DEGs in the livers of largemouth bass under HES or HET treatment by a heatmap. Red and blue colors indicate up- and down-regulated genes, respectively.

To investigate the molecular mechanisms underlying the response of *M. salmoides* to HS, we determined gene expression profiles in the livers of largemouth bass exposed to HS for different treatment periods. In the present study, a strict criterion of twofold difference and Padj < 0.05 were used (Figure 2a,b). Compared to the Con, under the HES treatment, 4811 genes, including 2207 up-regulated and 2604 down-regulated DEGs, were identified in the livers of largemouth bass from 6 biological replicates (Figure 2a,c and Table S3). In contrast, for the HET treatment, 3788 genes, including 1375 up-regulated and 2413 down-regulated DEGs, were detected in the livers of largemouth bass from 6 biological replicates (Figure 2b,c and Table S4). Because our main goal was to identify HS-responsive genes in largemouth bass livers, we combined the DEGs from both HES and HET treatments as HS-responsive genes. Accordingly, 6114 DEGs were identified in the livers of largemouth bass following different HS treatments, including 2645 up-regulated and 3469 down-regulated DEGs (Figure 2c and Table S5). Additionally, we performed heatmap analysis to determine the hierarchical clusters among these HS-responsive DEGs. As expected, these DEGs displayed substantial differences in the livers of largemouth bass under HS treatment (Figure 2d).

### 3.3. STEM Analysis of HS-Responsive DEGs in Livers of Largemouth Bass

**Figure 3:**
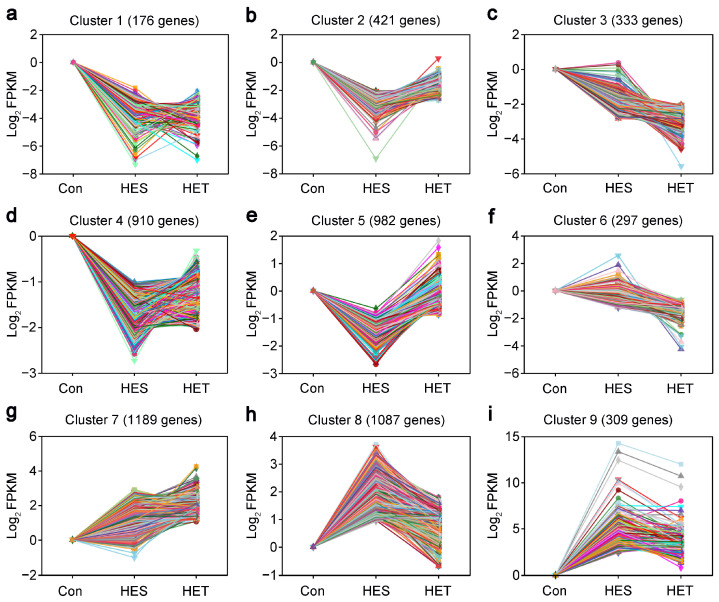
Temporal expression analysis of differentially expressed genes (DEGs) in the livers of largemouth bass (*M. salmoides*) of the heat-sensitive subgroup (HES) or heat-tolerant subgroup (HET) using STEM software (v1.3.13). (**a**–**i**) The DEGs were classified into nine main clusters according to the temporal gene expression patterns. The number of enriched genes belonging to each cluster is shown in parentheses. For each gene, ratios = Log_2_ (FPKM of the DEGs in HS samples/FPKM value of the DEGs in the control group). The minimum variation of gene expression levels between each treatment was twofold, with Padj < 0.05.

To gain more information on the expression patterns of these HS-responsive DEGs, we further performed a STEM analysis as described previously [40,51]. The results showed that these DEGs were mainly grouped into nine distinct temporal expression patterns following HS (Figure 3). The prominent profiles demonstrated that the expression patterns of most DEGs were quickly down-regulated by HES treatment (Figure 3a–d). These decreased expression patterns extended along the same trajectory until HET treatment (Figure 3a–d). Conversely, some DEGs were dramatically suppressed by HES treatment but were induced by HET treatment (Figure 3e). Furthermore, some DEGs were significantly induced by HES treatment but not by HET treatment (Figure 3f). Additionally, some DEGs were strongly induced by HES treatment and slightly induced by HET treatment (Figure 3g–i). Notably, 14 DEGs annotated as HSP genes were identified, 13 of which were markedly up-regulated following both HES and HET treatments (Figure 3f–i and Table S6). Together, these gene expression patterns suggest that there may be a time-specific response to HS treatment in the livers of largemouth bass.

### 3.4. Enrichment Analysis of HS-Responsive DEGs in Livers of Largemouth Bass

**Figure 4:**
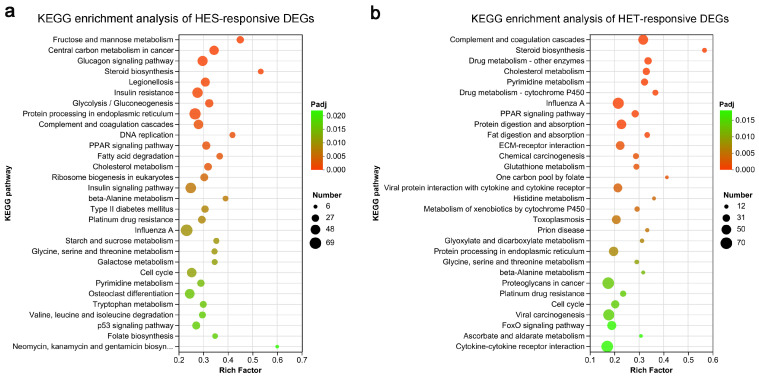
Enrichment analysis of Kyoto Encyclopedia of Genes and Genomes (KEGG) pathways of the differentially expressed genes (DEGs) in the livers of largemouth bass (*M. salmoides*) of the heat-sensitive (HES) subgroup or heat-tolerant (HET) subgroup using bubble charts. (**a**) The top 30 enriched KEGG pathways of DEGs under HES treatment. (**b**) The top 30 enriched KEGG pathways of DEGs under HET treatment. The dot colors indicate the Padj enrichment values, and the dot sizes represent the number of genes within each enriched pathway.

To further investigate the stress response of largemouth bass to HS, a KEGG enrichment analysis of the HES-responsive and HET-responsive DEGs was performed. Among these HES-responsive DEGs, 3500 were mapped to 343 predicted KEGG terms, of which 45 were highly enriched (Padj < 0.05). Notably, eight KEGG terms, including ‘fructose and mannose metabolism’, ‘central carbon metabolism in cancer’, ‘glucagon signaling pathway’, ‘steroid biosynthesis’, ‘legionellosis’, ‘insulin resistance’, ‘glycolysis/gluconeogenesis’, and ‘protein processing in endoplasmic reticulum’, were significantly enriched using a threshold value (Padj < 0.001) (Figure 4a). In contrast, among these HET-responsive DEGs, 2659 could be assigned to 342 predicted KEGG pathways, of which 47 were significantly enriched (Padj < 0.05) (Figure 4b). Among these pathways, ‘complement and coagulation cascades’, ‘steroid biosynthesis’, and ‘drug metabolism—other enzymes’ were among the three most important pathways (Figure 4b). In addition, KEGG pathways such as ‘cholesterol metabolism’, ‘pyrimidine metabolism’, ‘drug metabolism—cytochrome P450’, ‘influenza A’, ‘PPAR signaling pathway’, ‘protein digestion and absorption’, and ‘fat digestion and absorption’ also showed significant changes following HS (Padj < 0.001) (Figure 4b). These results indicate that these KEGG metabolic pathways are potentially involved in the stress response of largemouth bass to HS.

**Figure 5:**
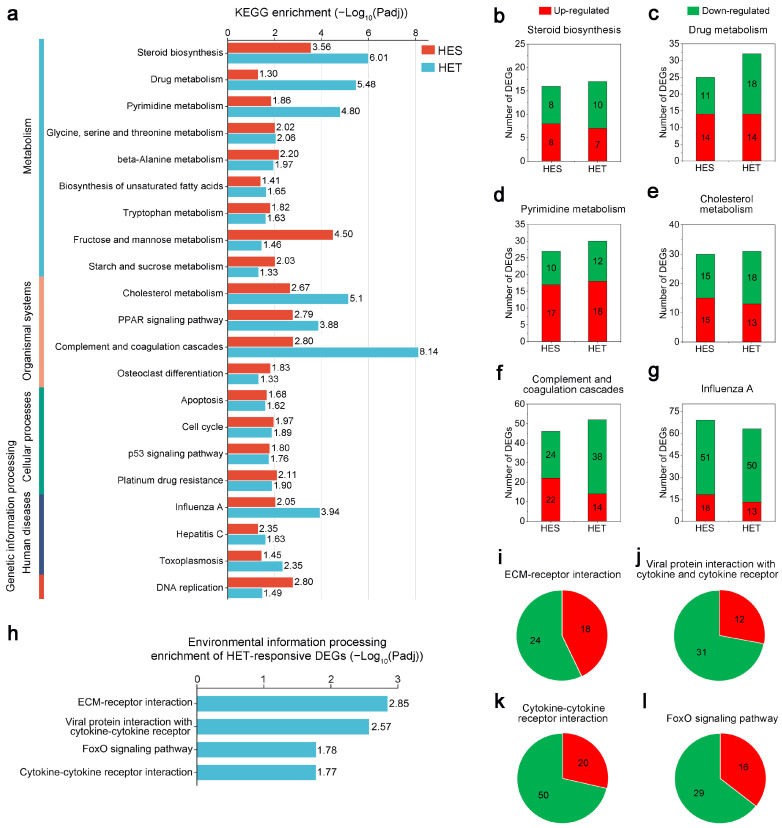
Effects of heat stress on Kyoto Encyclopedia of Genes and Genomes (KEGG) pathways of largemouth bass (*M. salmoides*). (**a**) KEGG pathways identically enriched for the differentially expressed genes (DEGs) in the livers of largemouth bass from the heat-sensitive (HES) subgroup or heat-tolerant (HET) subgroup. The abscissa presents the statistical significance (−Log10-transformed corrected Padj) of the KEGG terms following HES and HET treatments. Red and blue represent HES and HET, respectively. (**b**–**g**) The number of up- and down-regulated DEGs with the most significant KEGG terms following HES and HET treatments. ‘Steroid biosynthesis’ (**b**), ‘Drug metabolism’ (**c**), ‘Pyrimidine metabolism’ (**d**), ‘Cholesterol metabolism’ (**e**), ‘Complement and coagulation cascades’ (**f**), and ‘Influenza A’ (**g**) terms. (**h**) Enrichment analysis of KEGG pathways of HET-responsive DEGs involved in environmental information processing. The abscissa presents the statistical significance (−Log10-transformed corrected Padj) of the KEGG terms. (**i**–**l**) The number of up- and down-regulated DEGs involved in environmental information processing. ‘ECM-receptor interaction’ (**i**), ‘Viral protein interaction with cytokine and cytokine receptor’ (**j**), ‘Cytokine–cytokine receptor interaction’ (**k**), and ‘FoxO signaling pathway’ (**l**) terms.

Next, we investigated the differential effects of HS on the liver of largemouth bass at the first and second levels of the KEGG categories. Significant Padj values of the HES- and HET-responsive DEGs for each KEGG term were calculated. Under HES and HET conditions, these KEGG terms were clustered into five categories and are indicated by different colored bars in Figure 5a. In the metabolism category, DEGs belonging to ‘steroid biosynthesis’, ‘drug metabolism’, and ‘pyrimidine metabolism’ were differentially changed after HES and HET treatments. In the organismal systems category, DEGs belonging to ‘cholesterol metabolism’ and ‘complement and coagulation cascades’ were differentially changed after HES and HET treatments. In the human diseases category, DEGs belonging to the ‘influenza A’ term were differentially changed after HES and HET treatments. Notably, except for the ‘influenza A’ pathway, the number of DEGs related to ‘steroid biosynthesis’, ‘drug metabolism’, ‘pyrimidine metabolism’, ‘cholesterol metabolism’, and ‘complement and coagulation cascades’ under the HES treatment was more significant than that under the HET treatment (Figure 5b–g).

Interestingly, in the environmental information processing category, only DEGs from the HET subgroup were identified and grouped into four KEGG terms: ‘ECM-receptor interaction’, ‘viral protein interaction with cytokine and cytokine receptor’, ‘cytokine–cytokine receptor interaction’, and ‘FoxO signaling pathway’ (Figure 5h). Most DEGs implicated in these pathways were down-regulated by HET treatment (Figure 5i–l).

### 3.5. GGI and Expression Analyses of DEGs Involved in ECM-Receptor Interaction Pathway in Largemouth Bass

**Figure 6:**
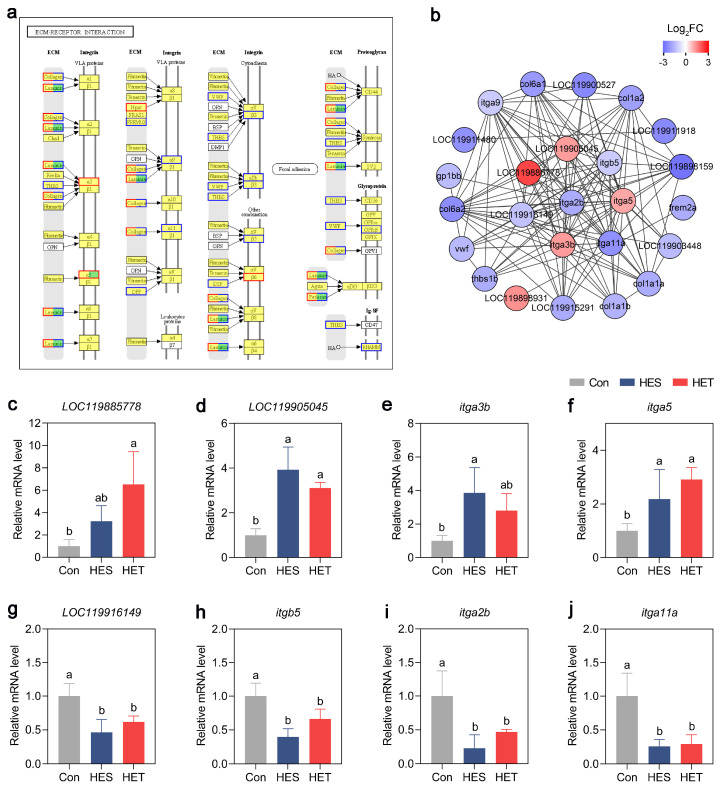
Gene–gene interaction (GGI) and expression analyses of the differentially expressed genes (DEGs) in livers of largemouth bass (*M. salmoides*) involved in the ECM-receptor interaction pathway following heat stress (HS). (**a**) Regulatory changes in the pathway of ECM-receptor interaction under HS treatments. The red and blue boxes indicate up- and down-regulated DEGs, respectively. (**b**) GGI networks of the DEGs involved in the ‘ECM-receptor interaction’ pathway. The scale from blue to red represents a gradual increase in values of Log_2_ transformed fold change ratios of the DEGs. (**c**–**j**) Quantitative reverse transcription PCR (qRT-PCR) data for the mRNA expression levels of the DEGs implicated in the ‘ECM-receptor interaction’ pathway in the livers of largemouth bass under HS treatments. The values represent the relative mRNA levels and are normalized to the mean of the control group. Data are given as the mean of three independent biological replicates ± standard deviation (SD). Lowercase letters indicate significant differences (Tukey’s test; *p* < 0.05).

As mentioned above, the ‘ECM-receptor interaction’, which is an essential adaptive pathway in animals under stress conditions, was ranked number one following HET treatment (Figure 5h). Therefore, we further illustrated this pathway and analyzed the GGI networks of the ‘ECM-receptor interaction’ pathway-related DEGs (Table S7). As a result, 42 DEGs covering 17 components of this pathway were identified (Figure 6a). Twenty-five DEGs in the ‘ECM-receptor interaction’ pathway interacted with each other, especially eight DEGs—*LOC119885778*, *LOC119905045*, *itga3b*, *itgb5*, *LOC119916149*, *itga5*, *itga2b*, and *itga11a* (Figure 6b). Thus, we further examined this pathway by determining the expression levels of the eight hub genes belonging to the ‘ECM-receptor interaction’ pathway using qPCR. The results revealed that the expression levels of *LOC119885778*, *LOC119905045*, *itga3b*, and *itgb5* were significantly up-regulated by more than twofold following HES treatment, and these up-regulation patterns continued to HET treatment (Figure 6c–f). In contrast, the expression levels of the other four hub genes, *LOC119916149*, *itga5*, *itga2b*, and *itga11a*, were markedly down-regulated after HES and HET treatments (Figure 6g–j). These qPCR results agreed with the transcriptomic data (Figure 2 and Figure 3 and Table S5). These results suggest that significant changes in genes involved in the ‘ECM-receptor interaction’ pathway indicate an alteration of ECM-receptor interaction in the livers, which can be used by largemouth bass to cope with HS.

## 4. Discussion

It has been shown that temperature is one of the critical environmental factors that affect the growth, development, and survival of aquatic organisms [12,52,53,54]. With the intensification of global warming, water temperatures are frequently higher than optimal for most aquatic animals during summer [31,55]. Over the past two decades, high temperatures have become a common limitation in aquatic ecosystems and have attracted extensive attention worldwide [56,57,58]. In China, HS is considered a severe threat to aquaculture animals, particularly subtropical fish, which have rigorous optimal growth temperatures in their aquatic environments [12,13,59,60]. Therefore, it is imperative to investigate the molecular response mechanisms of subtropical fish to HS.

Over the past decade, the availability of transcriptomic data from fish in response to HS has dramatically expanded. Data sets from the spleen and blood tissues of *Ctenopharyngodon idellus* subjected to HS with 3355 and 260 DEGs were produced by Yang et al. [59] and Huang et al. [7], respectively. Data sets from the liver and head kidney tissues of *Oncorhynchus mykiss* following HS with 128 and 443 DEGs, respectively, were generated by Li et al. [60] and Huang et al. [61]. Data sets from the livers of *Salmo salar* upon HS with 331 and 1900 DEGs were identified by Shi et al. [17] and Beemelmanns et al. [15], respectively. A data set from the gonad of *Cynoglossus semilaevis* under HS with 3702 DEGs was reported by Wang et al. [62]. A data set from the liver of *Scophthalmus maximus* in response to HS with 2067 DEGs was published by Zhao et al. [63]. Data sets from the liver and brain tissues of *Ctenopharyngodon idella* under HS with 2534 and 1622 DEGs, respectively, were identified by Zhang et al. [64]. A data set from the liver of *Larimichthys polyactis* following HS with 5328 DEGs was determined by Liu et al. [8]. Finally, a data set from the gill of *Acipenser baerii* upon HS with 8570 DEGs was reported by Yang et al. [65]. Consistently, we found that HS-responsive DEGs were predominantly involved in protein metabolism, energy metabolism, and immune systems in the present study.

In this study, an RNA-seq-based comparative transcriptomic analysis of HS-treated and control *M. salmoides* livers was carried out to explore the response mechanisms of largemouth bass to HS. A total of 6114 DEGs, which included 2645 up-regulated and 3469 down-regulated genes, were identified in *M. salmoides* livers under HS treatment (Figure 2). This result suggests that a large number of transcriptional alterations occur during HS treatment. This finding corroborates previous studies showing that fish undergo extensive gene changes in their livers in response to HS [8,15,17,63,64]. STEM analysis showed that 6114 DEGs were grouped into 9 distinct temporal expression patterns under HS treatment (Figure 3). KEGG enrichment analysis demonstrated that the HES-responsive DEGs were predominantly involved in ‘fructose and mannose metabolism’, ‘central carbon metabolism in cancer’, ‘glucagon signaling pathway’, ‘steroid biosynthesis’, ‘legionellosis’, ‘insulin resistance’, ‘glycolysis/gluconeogenesis’, and ‘protein processing in endoplasmic reticulum’, and the HET-responsive DEGs were mainly implicated in ‘complement and coagulation cascades’, ‘steroid biosynthesis’, ‘drug metabolism—other enzymes’, ‘cholesterol metabolism’, ‘pyrimidine metabolism’, ‘drug metabolism—cytochrome P450’, ‘influenza A’, ‘PPAR signaling pathway’, ‘protein digestion and absorption’, and ‘fat digestion and absorption’ (Figure 4), suggesting that these KEGG pathways might be implicated in the response of largemouth bass to HS.

Interestingly, an ‘ECM-receptor interaction’ pathway belonging to the environmental information processing category was identified as specific to HET treatment (Figure 5h). As shown in Figure 6a, 42 DEGs were assigned to the ‘ECM-receptor interaction’ pathway. Furthermore, GGI analysis identified eight DEGs as hub genes in the livers of largemouth bass in response to HS (Figure 6b). These data indicate that the ‘ECM-receptor interaction’ pathway in the livers of largemouth bass plays a potential role in responding to HS. To further determine the precise role of the ‘ECM-receptor interaction’ pathway, we examined the expression levels of hub genes in the livers of largemouth bass following HS. Compared to the Con, these hub genes showed differential expression profiles in response to HES and HET treatments (Figure 6c–g). These results suggest that the ‘ECM-receptor interaction’ pathway in the livers of largemouth bass has a crucial role in the response of *M. salmoides* to HS. This finding is consistent with previous studies showing that the ‘ECM-receptor interaction’ pathway can be strongly induced in fish in response to HS [59,66,67].

**Figure 7:**
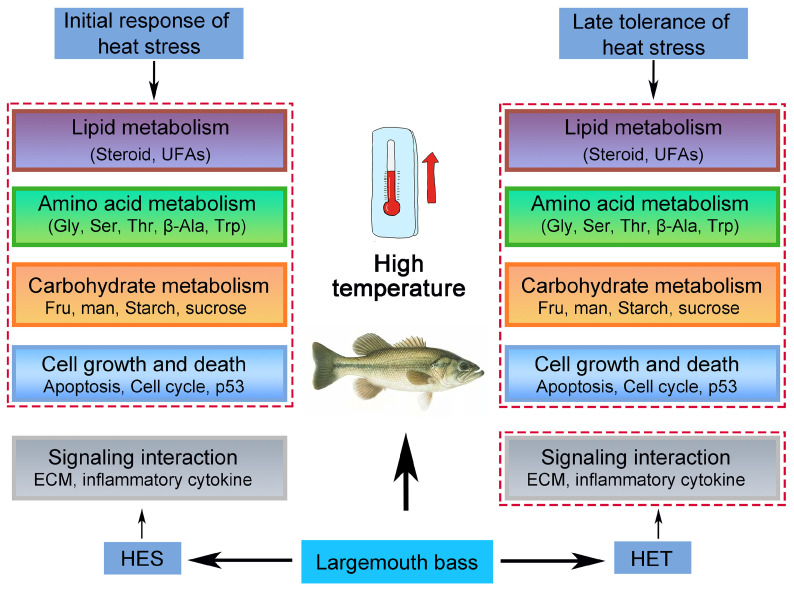
A proposed model of the heat stress response of largemouth bass (*M. salmoides*) to high temperatures. When faced with high temperatures, largemouth bass adapt to heat stress (HS) by modifying the metabolism of lipids, sugars, and amino acids, as well as the immune system. Abbreviations are: Steroid: steroid metabolism; UFAs: unsaturated fatty acids metabolism; Gly, Ser and Thr: glycine, serine and threonine metabolism; β-Ala; β-alanine metabolism; Trp: tryptophan metabolism; p53: p53 signaling pathway; ECM: ECM-receptor interaction; inflammatory cytokine: cytokine–cytokine receptor interaction and viral protein interaction with cytokine–cytokine receptor. The red dashed-line boxes indicate the changed pathways under HS.

Based on our results and those of previous studies [12,28,68], a possible regulatory model can be proposed for largemouth bass in response to HS. As illustrated in Figure 7, at high temperatures, HS causes dramatic changes in lipid, amino acid, and carbohydrate metabolisms, ultimately resulting in cell growth restriction and cell death. These alterations may help largemouth bass cope with HS and adapt to high-temperature conditions. However, we also found that largemouth bass responded differently to HS at different treatment times. More DEGs were found under the HES treatment than the HET treatment. In contrast, under the HET treatment, signaling interaction pathways in the environmental information processing were significantly altered in the livers of largemouth bass (Figure 7). This may be a strategy used by largemouth bass to adapt to HS under the HET treatment, in which a dynamic balance between heat tolerance and normal growth allows them to adapt more quickly to the external environment.

## 5. Conclusions

We performed a comparative transcriptomic analysis of the livers of largemouth bass. The transcriptome results showed profound transcriptional changes in the livers of largemouth bass following HS. KEGG and GGI analyses revealed that ‘ECM-receptor interaction’ pathway-related genes were markedly changed under HS treatments. These results provide clues for understanding the stress responses of genes and networks to HS and expand our current understanding of the effects of high temperatures on gene expression in the livers of largemouth bass.

## Data Availability

All supporting data are included within the main article, and all supporting data are included within the main article and its Supplementary Files.

## Supplementary Materials

The following supporting information can be downloaded at: https://www.mdpi.com/article/10.3390/genes14112096/s1, Table S1: List of primers used in this study; Table S2: Summary of RNA-sequencing of the livers of largemouth bass (*M. salmoides*) following heat stress; Table S3: Summary of the differentially expressed genes (DEGs) in the livers of the heat-sensitive subgroup (HES) of largemouth bass (*M. salmoides*) following heat stress; Table S4: Summary of the differentially expressed genes (DEGs) in the livers of the heat-tolerant subgroup (HES) of largemouth bass (*M. salmoides*) following heat stress; Table S5: Summary of the differentially expressed genes (DEGs) in the livers of largemouth bass (*M. salmoides*) following heat stress; Table S6: Summary of the differentially expressed genes (DEGs) annotated as heat shock protein (HSP) genes in the livers of largemouth bass (*M. salmoides*) following heat stress; Table S7: Summary of the differentially expressed genes (DEGs) involved in the ‘ECM-receptor interaction’ pathway in the livers of largemouth bass (*M. salmoides*) following heat stress.
